# Type‐A Aortic Dissection Evading Aortic Dissection Detection Score in Vulnerable Patient: A Case Report

**DOI:** 10.1002/ccr3.70684

**Published:** 2025-09-02

**Authors:** Felix Wehking, Jan‐Christoph Lewejohann, Florian Buerckenmeyer

**Affiliations:** ^1^ Department of Emergency Medicine Jena University Hospital Jena Germany; ^2^ Department of Radiology Jena University Hospital Jena Germany

**Keywords:** aorta, emergencies, tomography, ultrasonography, vascular surgical procedures

## Abstract

Algorithms and tools are frequently utilized in emergency medicine workflows. Focusing on selective information, they are at risk of missing patients with atypical presentations of sometimes life‐threatening conditions. This case report highlights a female patient in her late 70s who was transferred to the emergency department due to vomiting and diarrhea after eating raw fish for lunch. While she was initially stable and symptom‐free, her dynamic course in the emergency department ultimately led to the diagnosis of an acute aortic dissection. After performing an emergency aortic arch replacement, the patient's postoperative stay and one‐year follow‐up were unremarkable. Concluding, this case report elaborates on the implications of respecting atypical presentations in vulnerable patients.

## Graphical abstract

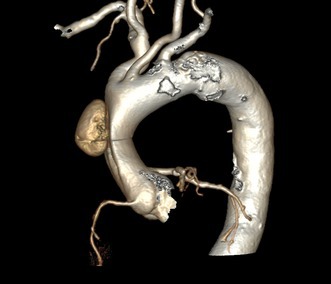

**Summary**

- Acute aortic dissections may present with atypical or nonspecific symptoms—even those suggestive of gastrointestinal infection.
- Time‐sensitive conditions may take time to uncover in the emergency department, highlighting the importance of re‐evaluating patients.

## 1 Introduction

Emergency medicine patients holding time‐sensitive pathologies may present with atypical or nonspecific symptoms, as repeatedly described in acute aortic dissection [1]. This case highlights an older female patient presenting with complaints suggestive of gastrointestinal infection who was suffering from acute aortic dissection. Due to the onset of additional symptoms in the emergency department, the aortic dissection could ultimately be diagnosed and treated.

## 2 History and Examination

A woman in her late 70s presented with an acute onset of vomiting and diarrhea after eating raw fish for lunch. She denied hematochezia, hematemesis, chest pain, headaches, neurologic symptoms, and syncope.

The patient's medical history revealed atrial fibrillation, a Warthin's tumor, and knee arthrosis. Furthermore, she visited the cardiology outpatient department regularly due to a bicuspid aortic valve. Her last echocardiography revealed a regular cardiac function without pathologies.

The initial vital signs were stable: Glasgow‐Coma‐Score 15, heart rate 86 per minute (atrial fibrillation), oxygen saturation 98% at rest in room air, respiratory rate 18 per minute, non‐invasive blood pressure 140 over 70 mmHg, temperature 36.9°C. The initial physical examination (abdomen, neurologic, heart and limbs) was unremarkable. More specifically, there was no heart murmur, no signs of heart failure, or neurologic deficits. The abdomen was soft on palpation. Peripheral pulse was palpable in all limbs.

## 3 Differential Diagnosis and Investigations

The patient's electrocardiogram, abdominal ultrasound, and blood gas analysis were unremarkable—apart from mild acidosis (pH 7.26) and elevated lactate of 4.9 mmol/L (cut‐off 2.2 mmol/L). Her blood sample revealed a leukocytosis of 17 × 10^9^/L (cut‐off 11.3 × 10^9^/L). Remaining blood parameters (inflammation, abdominal and renal function) were within normal range. Cardiac enzymes had not been requested at this time.

After 30 min in the emergency department and 1000 mL of intravenous full electrolyte solution, the patient was feeling well.

Given the history, symptoms, and unremarkable investigations, an acute gastrointestinal infection due to food poisoning appeared as the most likely explanation. Since the patient felt well after volume substitution, time‐critical pathologies like an atypical myocardial infarction or intestinal perforation were not considered. At this point, the treatment plan was vital sign observation over a period of 3 h and abdominal reassessment, followed by discharge. The increased lactate and leukocytosis were attributed to volume loss and physical stress.

After about 80 min in the emergency department, the patient became tachycardic (heart rate 160 per min, still in atrial fibrillation), reported an intermittent pain between her shoulder blades (“just like my chronic back pain”), and had difficulties breathing, which improved after leaning forward.

Re‐examination, electrocardiogram, and abdominal ultrasound were again inconclusive, and blood gas analysis remained unchanged—especially lactate (4.8 mmol/L). The most likely explanation was an acute worsening of her atrial fibrillation due to the suspected gastroenteritis. On the other hand, the increased lactate raised our suspicion for possible cardiac pathologies and perfusion deficit. After requesting high‐sensitive troponin, the point‐of‐care echocardiography revealed a circumferent pericardial effusion of about 1 cm, a preserved left ventricular function with mild mitral and aortic regurgitation, and a collapsing right ventricle during diastole. Combined with the present back pain and increased lactate, the patient was sent for immediate computed tomography, asking for aortic dissection.

**FIGURE 1:**
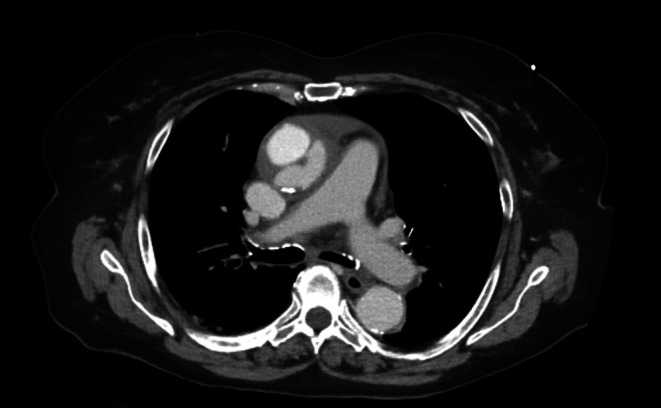
Axial computed tomography displaying Stanford type A/DeBakey type II acute aortic dissection.

**FIGURE 2:**
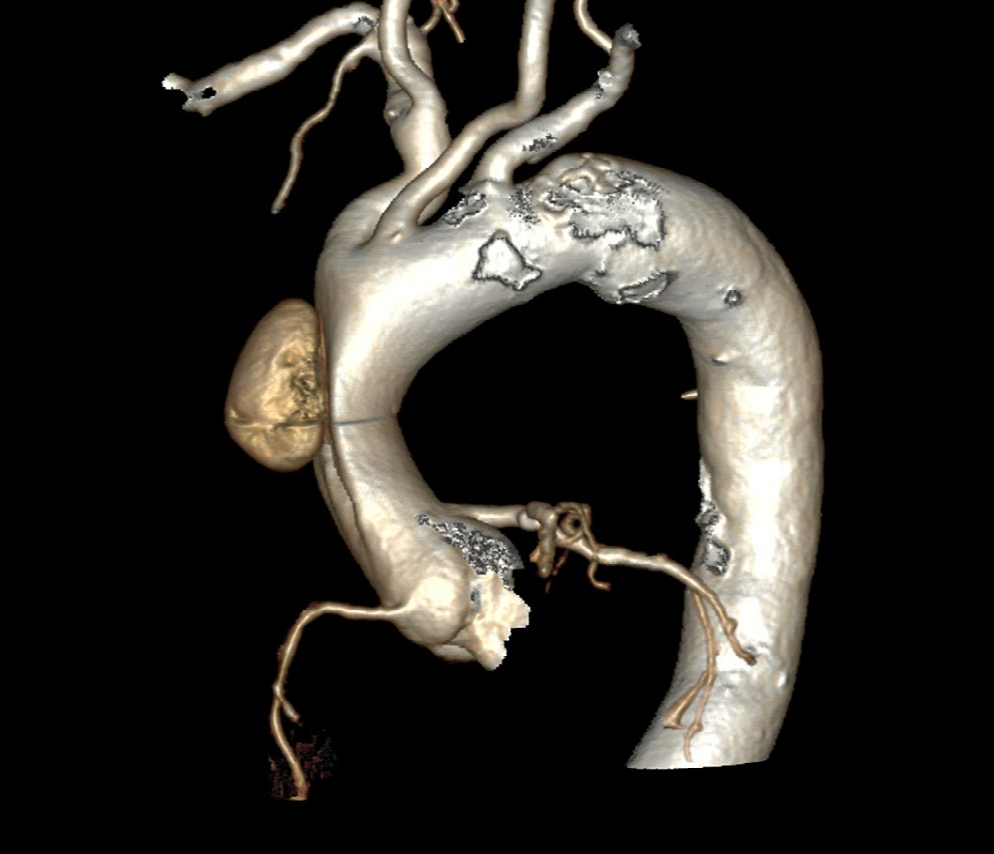
Computed tomography 3D‐reconstruction of the aortic arch displaying Stanford type A/DeBakey type II acute aortic dissection.

Radiology confirmed an acute aortic dissection of the ascending aorta without involvement of the aortic arch, coronary arteries, or the supraaortic vessels (Stanford Type A, DeBakey Type II) and a circular hemorrhagic pericardial effusion of 1.5 cm (Figures 1 and 2).

Communicating all results to cardiac surgery triggered immediate operative intervention.

## 4 Treatment

As the international normalized ratio was elevated under Phenprocoumon, prothrombin complex was substituted. Ultimately, the patient was transferred to the operating room after 140 min in the emergency department for supracoronary aortic arch replacement. The acute aortic dissection was confirmed during surgery. There were no intraoperative complications. The aortic valve, supra‐aortic vessels, and descending parts of the aorta were not affected by the dissection and therefore not replaced.

The postoperative stay was unremarkable. After seven days in the intensive care unit and nine days on the regular ward, the patient was discharged for rehabilitation without complications. Neither did major bleeding occur, nor was re‐exploration necessary.

## 5 Follow‐Up

In a phone call two months after the emergency department visit, the patient reported well‐being after discharge and rehabilitation. However, she still struggled walking longer distances (for instance, to the supermarket).

One year after the emergency department visit (during an appointment in the surgical outpatient department), the patient reported complete rehabilitation to her baseline. She did not need a mobilization device, and the follow‐up visit was unremarkable (clinical examination, laboratory, echocardiography and computed tomography).

There was no subsequent hospitalization during the one‐year follow‐up.

## 6 Discussion

This case highlights an initially atypical presentation of acute aortic syndrome. Current emergency medicine pathways suggest workup of several pathologies via pre‐test probability and validated scores. Yet, considering the established aortic dissection detection score, this patient's case would not initially suggest dissection (no syncope, no pain, no perfusion deficit in clinical examination). Even if aortic pathology was suspected, the patient would score 1 out of 3 points due to the bicuspid aortic valve and therefore suggest further workup via D‐Dimer [2].

Sorting this case into existing evidence, there are numerous reports of patients with acute aortic dissection presenting with atypical symptoms [3, 4, 5]. They include patients free of pain [3], having dyspnea as the main symptom [4] or showing aortic regurgitation in echocardiography [5]. These manifold presentations are also mentioned in broader research papers. For instance, the guidelines on aortic diseases by the American Heart Association and American College of Cardiology highlight chest and abdominal pain as frequent symptoms in acute aortic dissections [6]. The patient presented here was pain‐free. However, the guidelines also mention less specific presentations, including symptoms like nausea or dizziness [6].

Critics might argue that, while the initial presentation was atypical, the patient developed more typical symptoms during her emergency department stay. Hence, patients' re‐evaluation is one of this article's key messages.

Emergency physicians should be aware that pre‐test tools involve a substantial reduction of information, neglecting atypical presentations of time‐critical pathologies. In this case, the pathology was revealed through the dynamic course and reevaluation in the emergency ward. Especially women and older people are vulnerable patient groups, since their increased chance for atypical presentations is hardly reflected in some pre‐test tools.

## 7 Conclusions

Life‐threatening diseases like acute aortic dissection may present with initially nonspecific or atypical symptoms suggestive of gastrointestinal infection. Thus, established risk‐assessment tools might fail to detect them. Reevaluating emergency department patients increases the probability of detecting underlying, time‐sensitive conditions.

## Author Contributions

**Felix Wehking:** conceptualization, investigation, methodology, resources, visualization, writing – original draft, writing – review and editing. **Jan‐Christoph Lewejohann:** supervision, writing – review and editing. **Florian Buerckenmeyer:** data curation, investigation, writing – review and editing.

## Consent

Written informed consent was obtained from the patient for publication of this case report and any accompanying images.

## Conflicts of Interest

The authors declare no conflicts of interest.

## Data Availability

All relevant data are shared within the article.
